# Supplementary material for: Venomix: a simple bioinformatic pipeline for identifying and characterizing toxin gene candidates from transcriptomic data
Source: PeerJ. 2018 Jul 31;6:e5361. doi: 10.7717/peerj.5361 (PMC6074769; doi:10.7717/peerj.5361)
Supplement: Supplemental Information 3 [file peerj-06-5361-s003.gz › FinalOutput_E-20/C-type_lectin_mannose-binding_isoform_30/finaltree.pdf]

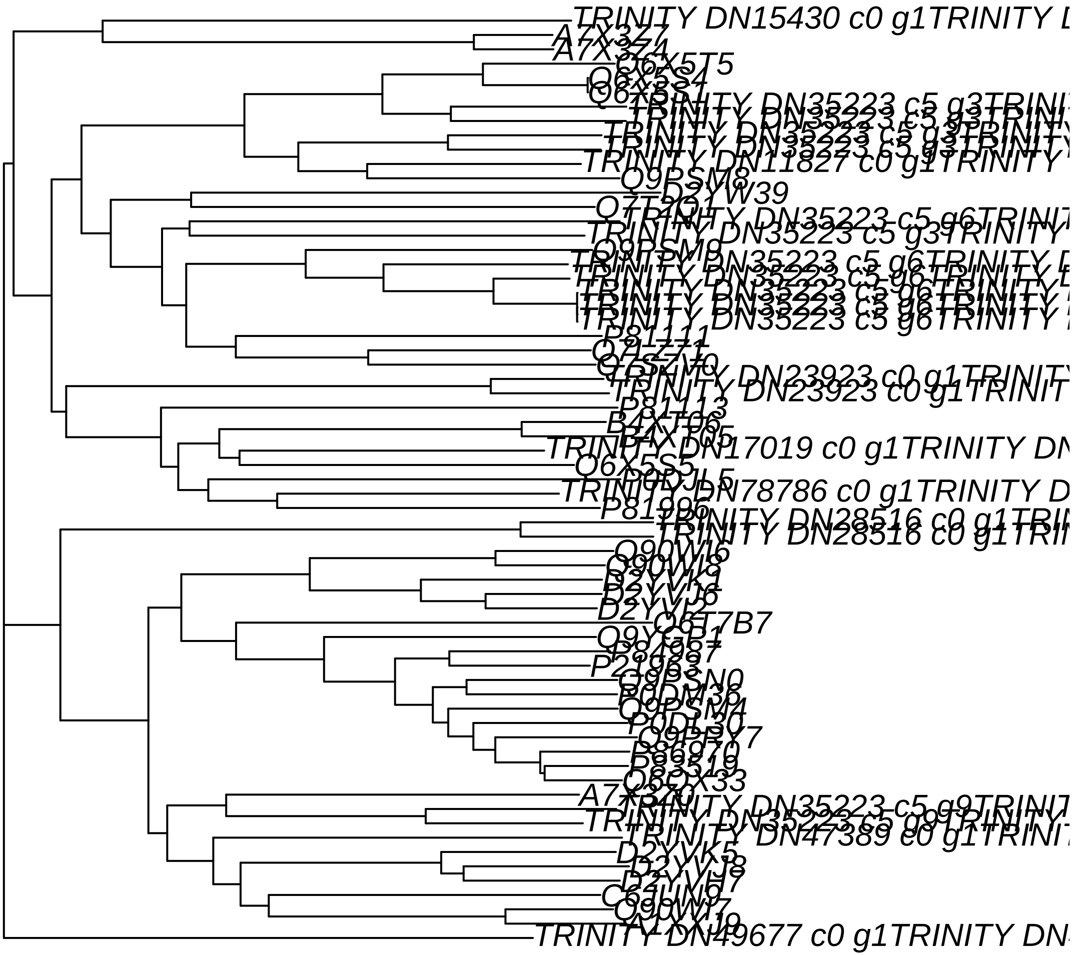

TRINITY DN15430 c0 g1TRINITY
A7X3Z7
A7X3Z1
Q6X5T5
Q6X5S4
Q6X5S1
TRINITY DN35223 c5 g3TRINITY
TRINITY DN11827 c0 g1TRINITY
Q9PSM8
D2YW39
TRINITY DN35223 c5 g6TRINITY
TRINITY DN35223 c5 g3TRINITY
Q9PSM9
P81111
TRINITY DN23923 c0 g1TRINITY
P81113
B4XT06
B4XT05
TRINITY DN17019 c0 g1TRINITY DN
Q6X5S5
TRINITY DN78786 c0 g1TRINITY D
P81996
TRINITY DN28516 c0 g1TRIN
D2YVJ6
Q6T7B7
P21987
P21983
Q9PSN0
P0DM36
Q9PSM4
P0DL30
Q9PXY7
P86970
P83519
Q8QX33
A7X3Z0
TRINITY DN35223 c5 g9TRINITY
TRINITY DN47389 c0 g1TRINIT
D2YVK5
D2YVJ8
D2YVH7
C6JUN9
Q90WI7
A1XXJ9
TRINITY DN49677 c0 g1TRINITY DN
